# Comparison of Effects of Sodium Bicarbonate and Sodium Carbonate on the Hydration and Properties of Portland Cement Paste

**DOI:** 10.3390/ma12071033

**Published:** 2019-03-28

**Authors:** Yuli Wang, Fengxia He, Junjie Wang, Qianku Hu

**Affiliations:** 1 School of Materials Science and Engineering, Henan Polytechnic University, Jiaozuo 454003, China; wangyuli@hpu.edu.cn (Y.W.); hefengxia321@163.com (F.H.); hqk@hpu.edu.cn (Q.H.); 2 Division of Engineering, New York University Abu Dhabi, Abu Dhabi P.O. Box 129188, UAE

**Keywords:** NaHCO_3_, Na_2_CO_3_, portland cement, compressive strength, setting time

## Abstract

Carbonates and bicarbonates are two groups of accelerators which can be used in sprayed concrete. In this study, the effects of the two accelerators sodium carbonate (Na_2_CO_3_) and sodium bicarbonate (NaHCO_3_) (0%, 1%, 2%, 3%, and 4% by weight of ordinary Portland cement OPC) on the properties of OPC paste were compared. The results show that both of them could accelerate the initial and final setting time of OPC paste, but the effect of the two accelerators on the compressive strength were different. After 1 day, sodium bicarbonate at 3% had the highest strength while sodium carbonate at 1% had the highest strength. After 7 days, both of the two accelerators at 1% had the highest compressive strength. After 28 days, the compressive strength decreased with the increase of the two. The improved strength at 1 and 7 days was caused by the accelerated formation of ettringite and the formation of CaCO_3_ through the reactions between the two with portlandite. The decrease of strength was caused by the Na^+^ could reduce the adhesion between C-S-H gel by replacing the Ca^2+^. NaHCO_3_ was found be a better accelerator than Na_2_CO_3_.

## 1. Introduction

Rapid hardening ability and high early strength are essential properties for shotcrete or sprayed concrete. Different accelerators were usually used in order to meet these requirements [1,2,3,4,5,6,7,8,9]. The mostly used accelerators including alkali carbonates, alkali hydroxide, alkali silicate, and alkali aluminate. For example, the sodium silicate was found to be able to modify the ITZ between the cement paste and aggregates and decrease the porosity of mortar [10]. Sodium aluminate and potassium aluminate can accelerate the formation of ettringite in the cement paste, and thus cause a rapid hardening effect [11]. Sodium aluminate was reported to be able to modify the pore structure of cement paste at an early age, improve the resistance to chloride ingress, and increase early-age compressive strength [12]. Carbonates [7,13,14,15,16,17] and bicarbonates with alkali were also among the most-used accelerators, such as Na_2_CO_3_ and NaHCO_3_ [18,19,20,21,22]. Mathur and Sharma [23] reported that the NaHCO_3_ can improve the strength and porosity of cement paste. Chandrawat and Yadav [24] found that the Na_2_CO_3_ could enhance the compressive strength and durability of cement paste. The work of Kunther et al. [25] showed that the bicarbonate ions could reduce the expansion of mortar and improve the sulfate resistance of mortar when subjected to sulfate attack. Yang et al. [26] reported that the addition of NaHCO_3_ and calcium lignosulphonate together could accelerate the formation of ettringite in the fly ash cement paste by changing the liquid-phase composition and the status of ettringite crystallization. Jang et al. [20] showed that the addition of 1% and 2% NaHCO_3_ could accelerate the hydration of cement paste and improve both flexural and compressive strength of the mortar specimen, but the addition of above 5% NaHCO_3_ caused the adverse effect on the strength development because of the formation of strong alkali NaOH in the specimen. Reddy et al. [27], however, reported that the both of Na_2_CO_3_ and NaHCO_3_ could decrease the compressive and tensile strength of concrete regardless of the content added or test age, and they also reported a significant reduction of strength after 28 days. However, Reddy and Krishna [28] reported that either Na_2_CO_3_ or NaHCO_3_ could increase the early age strength at 3 and 7 days but decrease significantly the strength after 28 days, besides, they reported that Na_2_CO_3_ accelerated the setting time whereas the NaHCO_3_ retarded the setting time. In addition, the structure and shape of the interface transition zone between the slurry and the aggregate in the cement composite material is a complicated problem. It has been well accepted that [29,30] the cement-based interface transition zone of the coarse aggregate is the weakest unit in the concrete, and the fly ash as mineral additive has a positive impact on the performance improvement of the interface transition zone.

It can be seen that there exist conflicted findings on the influence of Na_2_CO_3_ and NaHCO_3_ on the setting time and physical properties of cementitious materials. It is necessary to carry out a comprehensive study on the effects of the two accelerators on the properties of cement paste and make a comparison between the two. In order to investigate and compare the effects of the two accelerators on the properties of OPC paste, the same amount of Na_2_CO_3_ and NaHCO_3_ with 0%, 1%, 2%, 3%, and 4% weight of OPC were added into different mixes and the setting time and compressive strength at ages of 1, 7, and 28 days were studied, besides, the related hydration mechanism and hydration products were investigated through hydration heat, Thermogravimetry-Differential Thermal Analysis (TG-DTA), X-ray Diffraction (XRD), and Scanning Electron Microscopy (SEM) tests.

## 2. Materials and Methods

### 2.1. Materials

P.O. 42.5 ordinary Portland cement (OPC) in accordance with a Chinese standard GB175-2007 [31] was used. The physical properties and chemical composition of OPC are shown Table 1 and Table 2. The mineral composition of OPC is shown in Table 3. The NaHCO_3_ and Na_2_CO_3_ used were in powder form and the purity was >99.5% and >99.8%, respectively. A superplasticizer used was polycarboxylate. The mixing water was deionized water.

### 2.2. Methods

The mix design is shown in Table 4. Water-cement ratio (w/c) was kept same as 0.35. The superplasticizer was kept same as 0.5% by weight of OPC. OPC was firstly mixed with NaHCO_3_ or Na_2_CO_3_ in dry state. The water and superplasticizer were then added and mixing speed was at 60 rpm for 2 min followed by 120 rpm for another 2 min. The weight of NaHCO_3_ and Na_2_CO_3_ were added as 1%, 2%, 3%, and 4% by weight of cement in different mixes. Specimens were cured under s standard curing condition (20 °C, 97% R.H.).

The setting time of cement paste were tested according to a Chinese standard JC477-2005 [32]. A multichannel microcalorimeter was used for hydration heat test and it lasted for 24 h. Cubic samples with a dimension of 40 mm × 40 mm × 40 mm were used for compressive strength test. Compressive strength, TG-DTA, and SEM tests were conducted at ages of 1, 7, and 28 days. Powder samples were collected and the hydration was terminated by immersing into absolute ethyl alcohol for 24 h. The powder samples were then dried at 40 °C in a vacuum oven for 4 h. The samples were furtherly grounded by a pestle and mortar to pass a sieve with an aperture of 75 µm. The final powder samples were used for TG-DTA and XRD tests. The TG-DTA tests were conducted in a N_2_ environment with a simultaneous thermal analyzer system (HENVEN, Beijing, China) and the temperature was increased from 20 °C to 800 °C at a rate of 10 °C/min. Each time, one gram of the powder sample was used for XRD test. The XRD tests were conducted using a Rigaku SmartLab X-ray diffractometer (Rigaku, Tokyo, Japan) under a voltage of 40 kV and a current of 150 mA. The scanning rate was 10°/min from 5° to 70°. A MERLIN Compactfield Emission Scanning Electron Microscope (ZEISS, Jena, Germany) was used for SEM observations. The selected samples for SEM observations were at a size of around 5 mm in length and width with a fresh broken surface after the compression tests at ages of 1, 7, and 28 days. The samples were gold coated under vacuum condition before observation. The detailed procedures of hydration heat, compressive strength, TG-DTA, XRD, and SEM can also be found in our previous paper [33].

## 3. Results

### 3.1. Influence of NaHCO_3_/Na_2_CO_3_ on the Setting Time of OPC Paste

**Figure 1:**
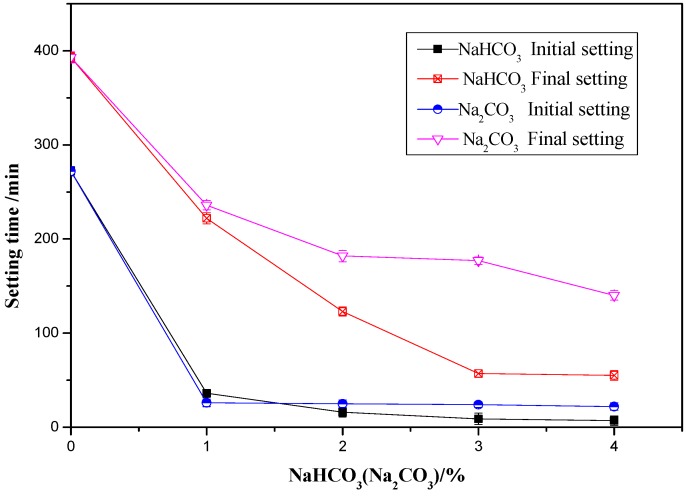
Effects of NaHCO_3_ and Na_2_CO_3_ on the setting time of OPC paste.

The influence of 0%, 1%, 2%, 3%, and 4% of NaHCO_3_/Na_2_CO_3_ on the initial and final setting time of OPC paste is shown in Figure 1. The results show that both of the initial and final setting time of the OPC paste decreased with the increase of NaHCO_3_ or Na_2_CO_3_ content. The initial setting time of OPC paste with 1%, 2%, 3%, and 4% NaHCO_3_ decreased by 86.76%, 94.12%, 96.69%, and 97.43% respectively compared to that of pure OPC paste. The final setting time of OPC paste with 1%, 2%, 3%, and 4% NaHCO_3_ decreased by 43.51%, 68.70%, 85.50%, and 86.01% respectively compared to that of pure OPC paste. It can be seen that the addition of 1–2% NaHCO_3_ significantly deceased the initial and final setting time of OPC paste. Further increase of NaHCO_3_ beyond 1% up to 4% showed little influence on the initial setting time, and further increase of NaHCO_3_ beyond 2% up to 4% showed little influence on the final setting time.

The initial setting time of OPC paste with 1%, 2%, 3%, and 4% Na_2_CO_3_ decreased by 90.44%, 90.80%, 91.18%, and 91.91% respectively compared to that of pure OPC paste. The final setting time of OPC paste with 1%, 2%, 3%, and 4% Na_2_CO_3_ decreased by 39.95%, 53.69%, 54.96%, and 64.38% respectively compared to that of pure OPC paste. It can be seen that the influence of Na_2_CO_3_ on the initial setting time was more significant than the final setting time. The Na_2_CO_3_ showed similar effect as NaHCO_3_ on the initial setting time but its influence on the final setting time was less than the NaHCO_3_. The related mechanisms will be discussed later.

### 3.2. Influence of NaHCO_3_/Na_2_CO_3_ on the Compressive Strength of OPC Paste

**Figure 2:**
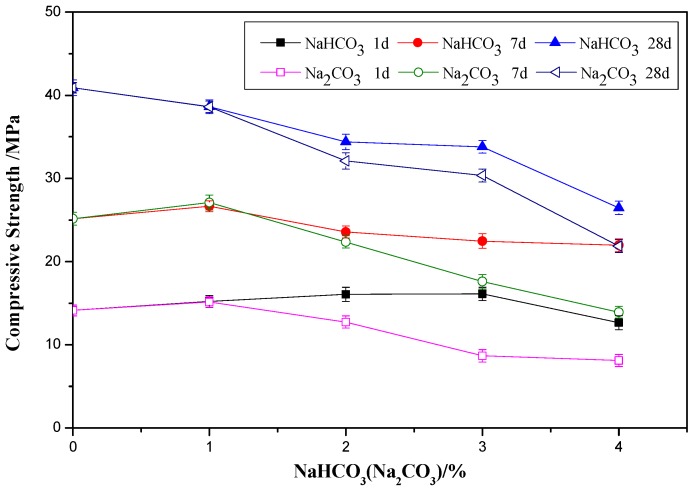
Effects of NaHCO_3_ and Na_2_CO_3_ on the compressive strength of OPC paste.

The effect of 0%, 1%, 2%, 3%, and 4% of NaHCO_3_ and Na_2_CO_3_ on the compressive strength of OPC paste specimen at the ages of 1, 7, and 28 days is shown in Figure 2. At the age of 1 day, with the increasing content of NaHCO_3_, the compressive strength of OPC paste increased initially and then decreased. The highest compressive strength happened in the mix with 3% NaHCO_3_. The strength of the mix with 3% NaHCO_3_ at the age of 1 day was 14% higher than that of the paste with no NaHCO_3_. At the age of 7 days, with the increase of NaHCO_3_, the compressive strength of OPC paste increased initially and then decreased with the highest strength happened in the mix with 1% NaHCO_3_. The strength of the mix with 1% NaHCO_3_ at the age of 7 days was 6% higher than that of OPC. At 28 days, the compressive strength of cement paste deceased continuously with the increase of NaHCO_3_. It can be seen that below 1% NaHCO_3_ can increase the early age strength but higher content of could decrease the later age strength significantly. This can be caused by the formation of NaOH [20], which is a strong alkali and could react with the silica sand in the paste specimen.

For the pastes with Na_2_CO_3_, the compressive strength at ages of 1 and 7 days firstly increased and then decreased with the increase of Na_2_CO_3_ content, and the paste with 1% Na_2_CO_3_ had the highest compressive strength. The compressive strength of paste with 1% Na_2_CO_3_ was 7.2% higher at age of 1 day and 7.7% higher at age of 7 days compared to that of OPC paste. Similarly to NaHCO_3_, the compressive strength of pastes with Na_2_CO_3_ at age of 28 days decreased continuously with the increase of Na_2_CO_3_. The reason could be that the formation of NaOH caused the decrease of compressive strength. From Figure 2 it can be seen that the NaHCO_3_ had the similar beneficial effect as Na_2_CO_3_ on the early age strength when the addition was below 1%, but much worse effect than Na_2_CO_3_ on the strength development when the addition was above 1%.

### 3.3. Hydration Heat

**Figure 3:**
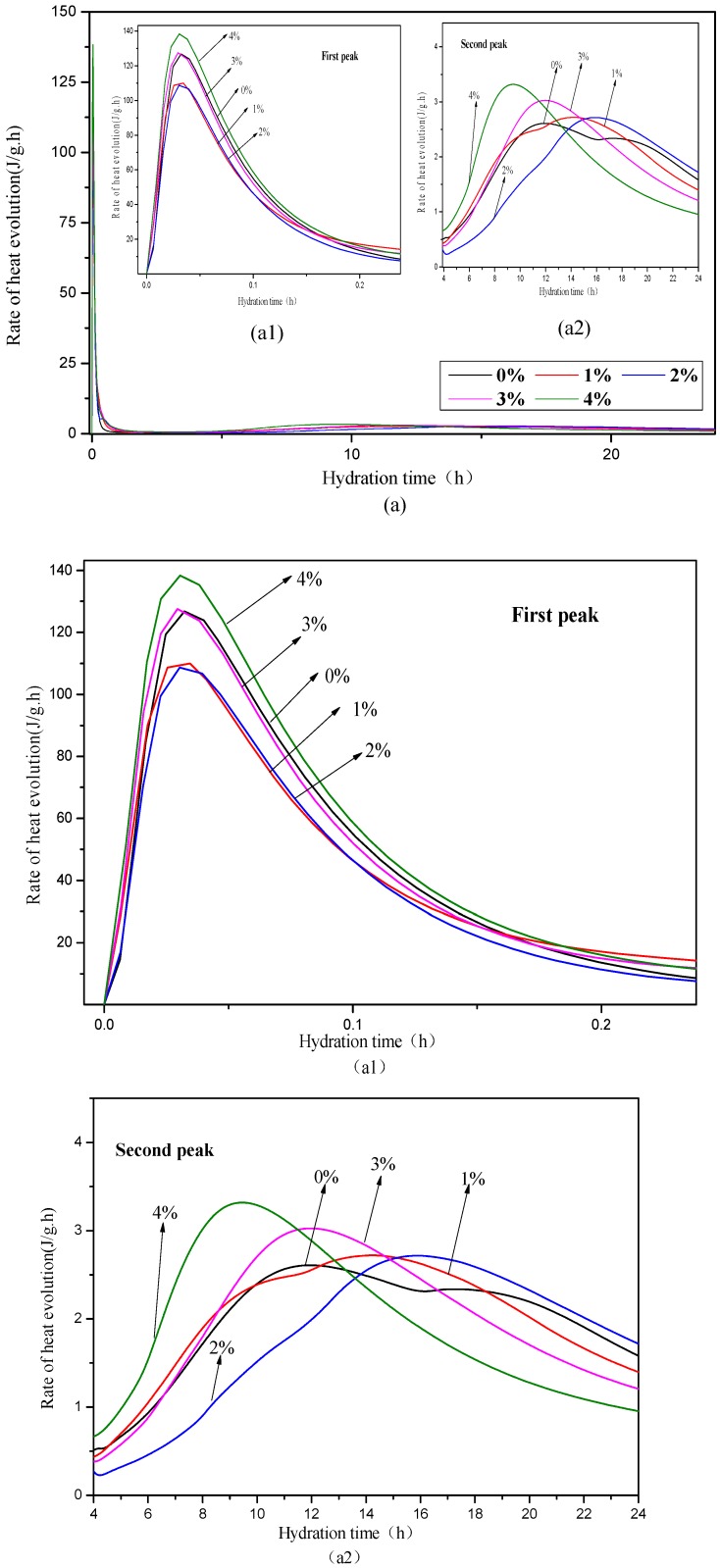

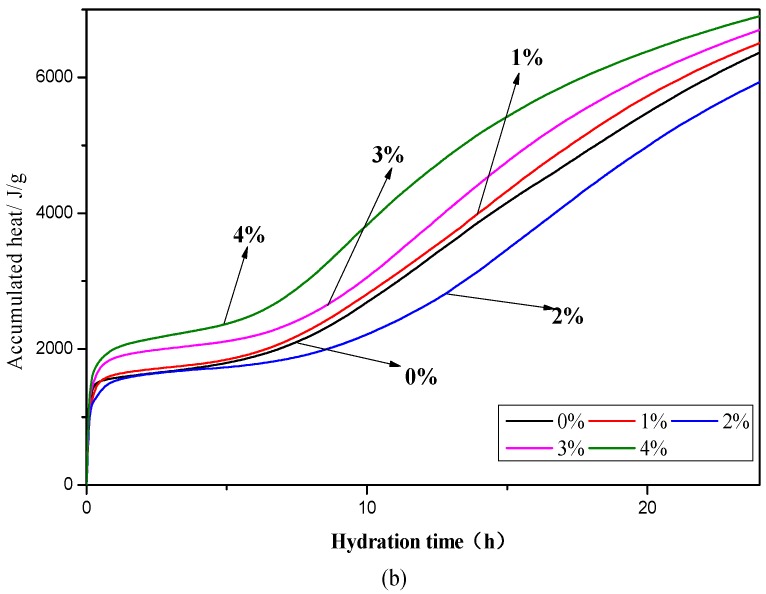
Hydration heat rate (**a**) and accumulated heat (**b**) of the pastes with different contents of NaHCO_3._

Figure 3 shows the hydration heat rate and accumulated hydration heat of the OPC pastes with 0%, 1%, 2%, 3%, and 4% NaHCO_3_. The first peak of hydration heat rate at around 0.05 h in Figure 3(a1) firstly decreased with the increase of NaHCO_3_ up to 2% and then increased with the further increase of NaHCO_3_ up to 4%. The first peak associated with the formation ettringite (AFt) [34,35], and it is suggested that the addition of 1–2% NaHCO_3_ refrained the formation of AFt in OPC paste but the further addition of NaHCO_3_ up to 4% accelerated the formation of AFt. The initial decrease of the AFt could be caused by the possible reaction or adhesion between the NaHCO_3_ and the aluminum phases, but the later increase of the AFt in the mix with 4% NaHCO_3_ could be caused by the increased CO_3_^2−^ content [17].

Different from the trend of the first peak of hydration heat rate with content of NaHCO_3_, as shown in Figure 3(a2), the peak height of the second peak of the hydration heat rate at 8–15 h increased continuously with the increase of NaHCO_3_. The second peak associated with the hydration of C_3_S and C_2_S and the formation of C-S-H and portlandite. It can be seen that the addition of increased the peak height of the second peak and it was indicated that there could be more hydration of C_3_S and C_2_S at 8–15 h in the mix with more NaHCO_3_. However, the peak time of the second peak was delayed in the mixes with 1% and 2% NaHCO_3_ but it was earlier in the mixes with 3% and 4% NaHCO_3_ compared to the control group with no NaHCO_3_. It suggested that the addition of NaHCO_3_ up to 2% delayed the hydration of C_3_S and C_2_S but further increase of NaHCO_3_ up to 4% accelerated the hydration of C_3_S and C_2_S in the initial 24 h.

The total accumulated hydration heat in the initial 24 h is shown in Figure 3b, and it shows that the difference between the total hydration heat of OPC paste and that of the mix with 1% NaHCO_3_ was not significant, the mix with 2% had a much lower hydration heat than the OPC paste, but the mixes with 3% and 4% had a significantly higher total hydration heat than the OPC paste. This was mainly caused by the previously described delayed effect on the hydration heat in the mix with 2% NaHCO_3_ and the accelerated effect on the hydration heat in the mixes with 3% and 4% NaHCO_3_.

**Figure 4:**
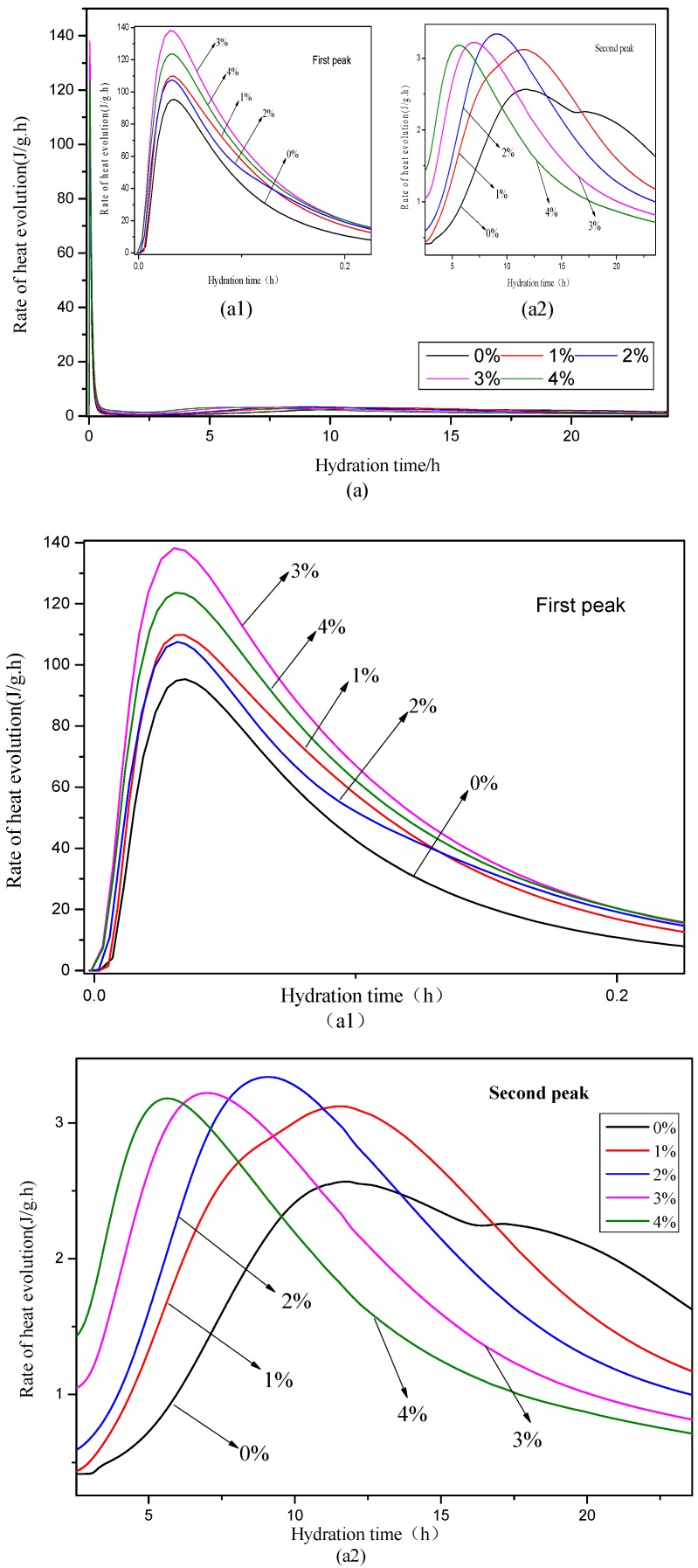

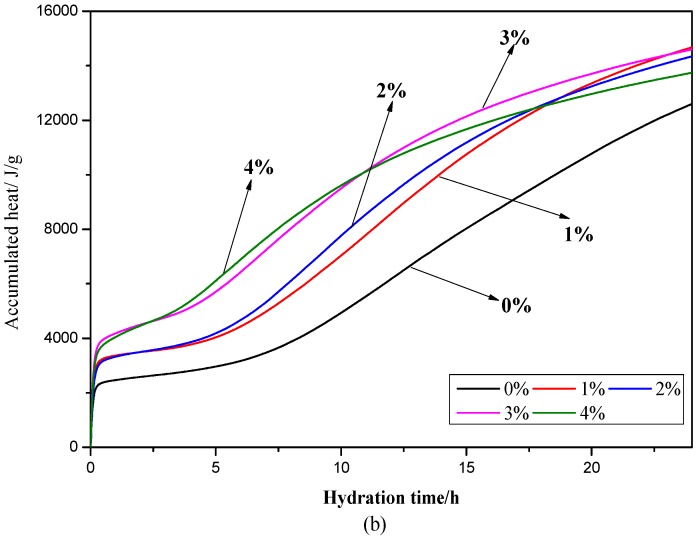
Hydration heat rate (**a**) and accumulated heat (**b**) of the pastes with different contents of Na_2_CO_3._

The hydration heat rate and accumulated hydration heat of the mixes with 0%, 1%, 2%, 3%, and 4% Na_2_CO_3_ are shown in Figure 4. The results show that the mixes with Na_2_CO_3_ had a higher first peak height of the hydration heat rate, as in Figure 4(a1), than the OPC paste with no Na_2_CO_3_. The highest first peak height happened in the mix with 3% Na_2_CO_3_ and there was a slightly decrease of the peak height in the mix with 4% Na_2_CO_3_. It is indicated that the addition of Na_2_CO_3_ accelerated the hydration of C_3_A and the formation of AFt.

As for the second peak, in Figure 4(a2), the addition of Na_2_CO_3_ increased the peak height and accelerated the peak time compared to the OPC paste with no Na_2_CO_3_. It suggested that the Na_2_CO_3_ accelerated and increased the hydration of C_3_S and C_2_S. This agrees with the findings in literature [33]. There was a shoulder peak at around 18 h after the second peak in the control group, which was cause by the secondary formation of AFt [36], but this shoulder peak did not appear in any mix with Na_2_CO_3_. This suggested that, in the mixes with Na_2_CO_3_, the initial accelerated formation of AFt in the first peak might consumed most of the C_3_A and formation of most AFt was finished at that time.

The accumulated hydration heat of the mixes with different contents of Na_2_CO_3_ is shown in Figure 4b. It can be seen that the mix with Na_2_CO_3_ had a much higher accumulated hydration heat compared to the control group. At 5–10 h, the mix with 4% Na_2_CO_3_ had the highest accumulated hydration heat and the higher content of Na_2_CO_3_ caused a higher accumulated hydration heat. At the end of 24 h, the mix with 1% Na_2_CO_3_ had the highest total hydration heat, followed by the mixes with 3%, 2%, 4%, and 0% Na_2_CO_3_. After 15 h, the increase rate of the accumulated hydration heat in the mixes with 3% and 4% Na_2_CO_3_ decreased obviously compared to the mixes with 1% and 2% Na_2_CO_3_. There was a tendency that the total hydration heat of the mixes with 3% and 4% Na_2_CO_3_ could be lower than the control group in the long term.

### 3.4. TG-DTA Results

**Figure 5:**
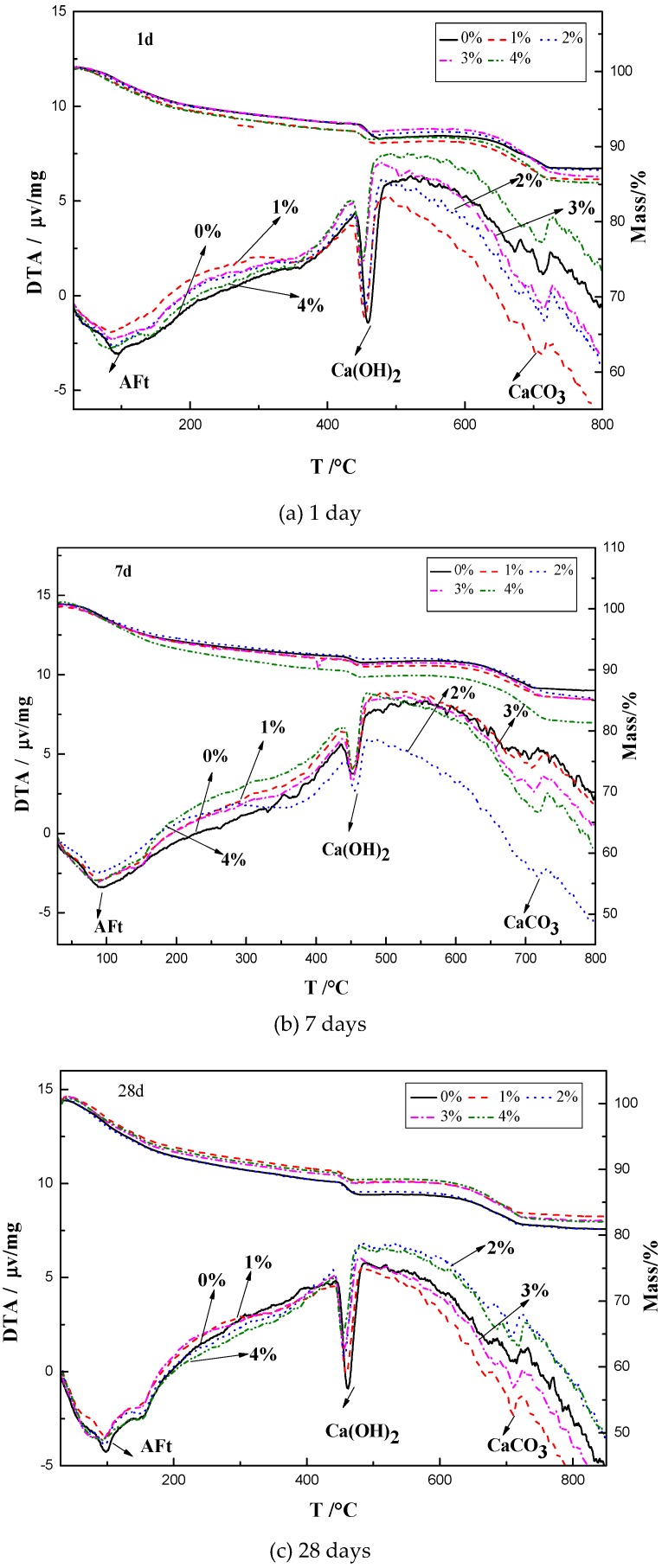
TG-DTA results of the pastes with different contents of NaHCO_3_ at ages of (**a**) 1, (**b**) 7, and (**c**) 28 days.

The TG-DTA results of the mixes with 0%, 1%, 2%, 3%, and 4% NaHCO_3_ are shown in Figure 5. There were three main DTA peaks at around 100 °C, 460 °C, and 700°C, which indicated the composition of AFt, portlandite and CaCO_3_ respectively. At the age of 1 day, the weight loss at the peak of AFt was 2.6%, 2.9%, 2.7%, 2.6%, and 3.1% in the mix with 0%, 1%, 2%, 3%, and 4% NaHCO_3_ respectively. The weight loss at the peak of portlandite was 2.0%, 1.6%, 1.5%, 1.2%, and 1.0% in the mix with 0%, 1%, 2%, 3%, and 4% NaHCO_3_ respectively. The weight loss at the peak of CaCO_3_ was 3.5%, 3.9%, 4.0%, 4.8%, and 4.7% in the mix with 0%, 1%, 2%, 3%, and 4% NaHCO_3_ respectively. It can be seen that the addition of NaHCO_3_ increased the formation of AFt and CaCO_3_ at the age of 1 day but decreased the portlandite. At the age of 7 and 28 days, the weight losses at the peaks of AFt, portlandite and CaCO_3_ showed similar trend as that at age of 1 day. It can be seen that the addition of NaHCO_3_ increased the formation of AFt and CaCO_3_ and decreased the portlandite at all the ages.

**Figure 6:**
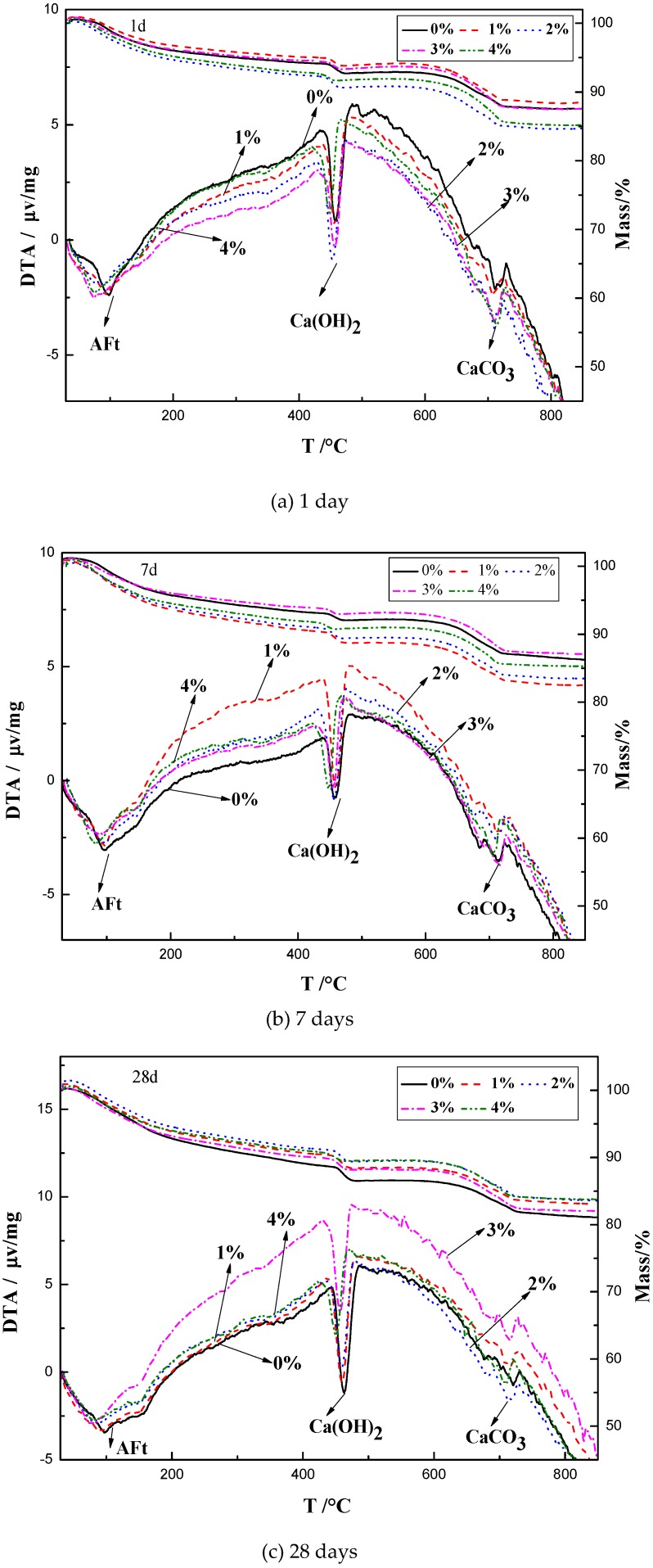
TG-DTA results of the pastes with different contents of Na_2_CO_3_ at ages of (**a**) 1, (**b**) 7, and (**c**) 28 days.

Figure 6 shows the TG-DTA results of the mixes with 0%, 1%, 2%, 3%, and 4% Na_2_CO_3_ at the ages of 1, 7, and 28 days. At the age of 1 day, the weight loss at the peak of AFt was 2.6%, 2.6%, 3.0%, 2.8%, and 3.0% in the mix with 0%, 1%, 2%, 3%, and 4% Na_2_CO_3_ respectively. The weight loss at the peak of portlandite was 2.0%, 1.6%, 1.4%, 1.1%, and 0.9% in the mix with 0%, 1%, 2%, 3%, and 4% Na_2_CO_3_ respectively. The weight loss at the peak of CaCO_3_ was 3.5%, 3.9%, 4.0%, 4.8%, and 4.7% in the mix with 0%, 1%, 2%, 3%, and 4% Na_2_CO_3_ respectively. It can be seen that, similar as the NaHCO_3_, the addition of Na_2_CO_3_ increased the formation of AFt and CaCO_3_ and decreased the portlandite at the age of 1 day. This trend was similar at the ages of 7 and 28 days.

These results showed that the influence of NaHCO_3_ on the formation of AFt, portlandite and CaCO_3_ was similar as Na_2_CO_3_. The mix with the highest amount of NaHCO_3_ or Na_2_CO_3_ had the highest amount of AFt and CaCO_3_ but the lowest amount of portlandite. As can be seen from Figure 5 and Figure 6, the addition of NaHCO_3_ or Na_2_CO_3_ made the overall weight loss of the blended paste higher than the control group at the age of 1 and 7 days but the lower than the control group at the age of 28 days.

### 3.5. XRD Results

**Figure 7:**
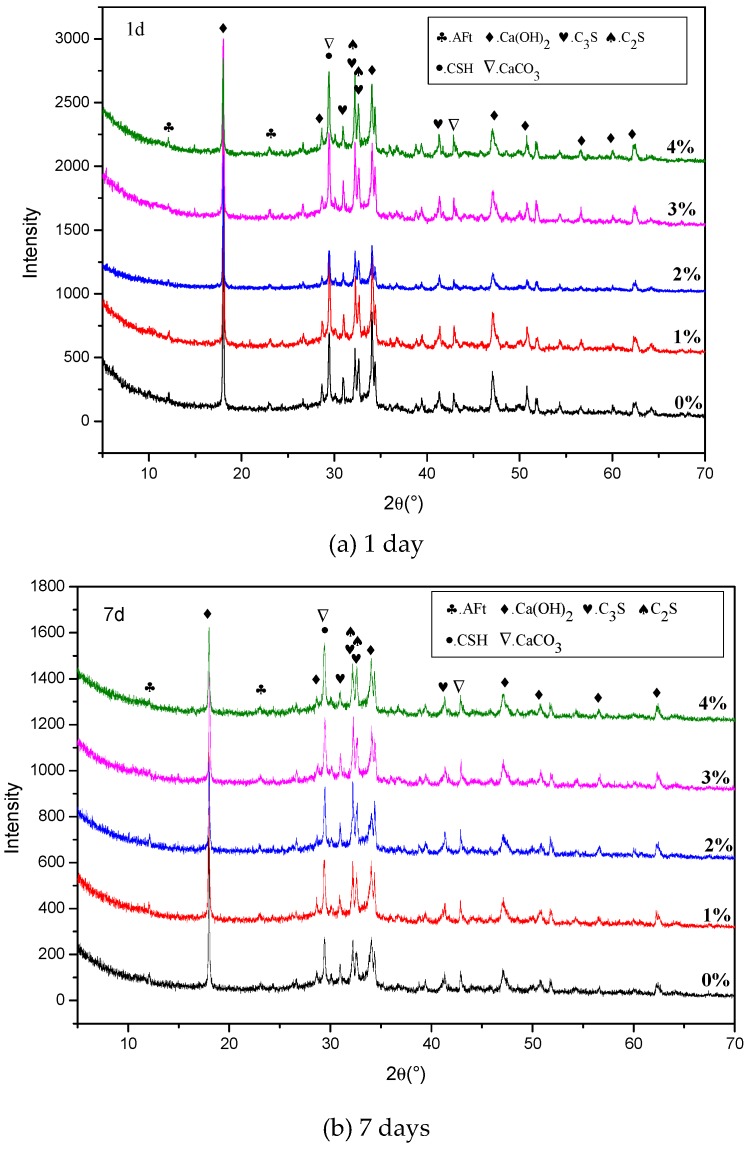

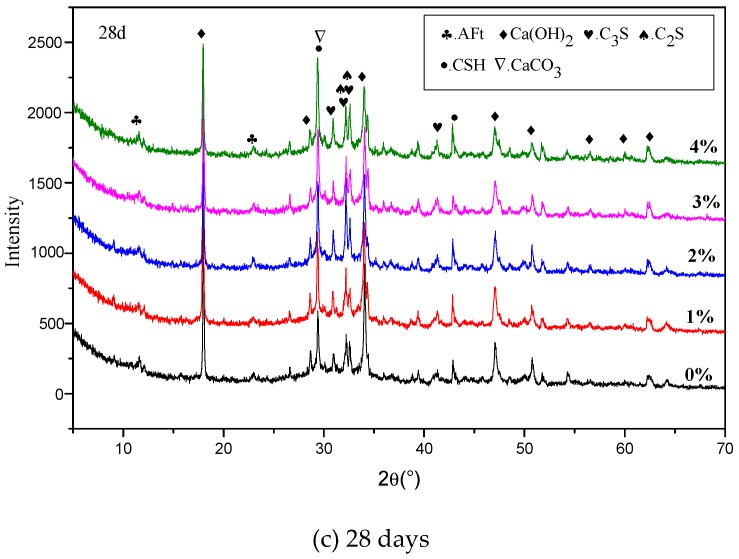
XRD spectrum results of the mixes with different contents of NaHCO_3_ at ages of (**a**) 1, (**b**) 7, and (**c**) 28 days.

The XRD results of the pastes with 0%, 1%, 2%, 3%, and 4% NaHCO_3_ at the ages of 1, 7, and 28 days are shown in Figure 7. It can be seen that, at the age of 1 day, the peaks of Ca(OH)_2_ at 2θ = 34° and 47° decreased with the increase of NaHCO_3_. At the ages of 7 and 28 days, the peaks of portlandite changed in the same way as that in 1 day, besides, the peak of C-S-H and CaCO_3_ at 2θ = 29° increased with the increase of NaHCO_3_. These results all agree with the previously reported findings in the TG-DTA results. The change of AFt in the XRD spectrum was not obvious for the mixes with different contents of NaHCO_3_.

**Figure 8:**
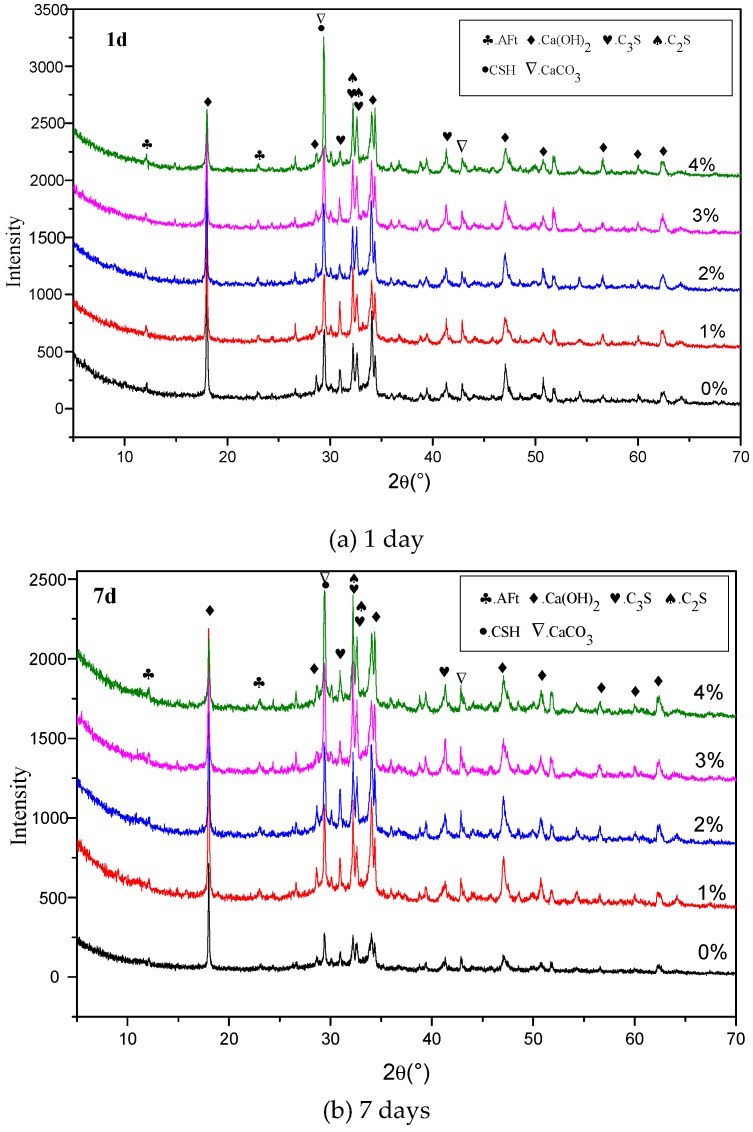

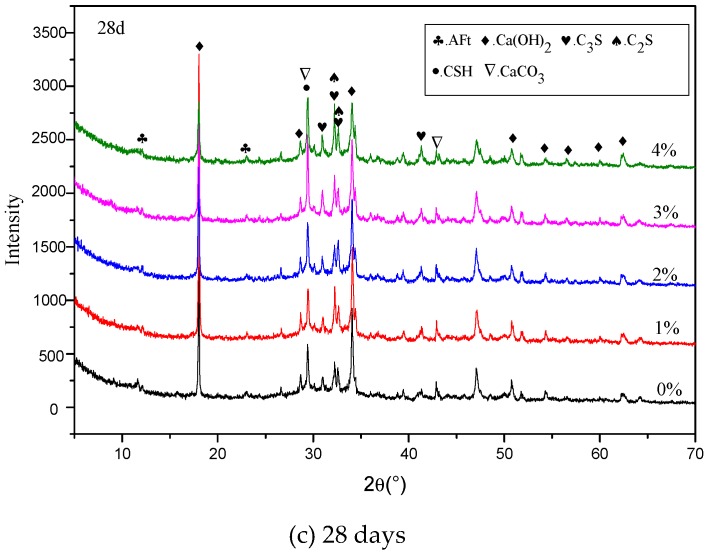
XRD spectrum results of the mixes with different contents of Na_2_CO_3_ at ages of (**a**) 1, (**b**) 7, and (**c**) 28 days.

Figure 8 shows the XRD results of the pastes with 0%, 1%, 2%, 3%, and 4% Na_2_CO_3_ at the ages of 1, 7, and 28 days. The results show that the peaks of C-S-H and CaCO_3_ increased with the increase of Na_2_CO_3_ content at all ages, at the same time, the portlandite decreased gradually with the increase of Na_2_CO_3_ content. This again agrees with the findings in TG-DTA results.

### 3.6. SEM Results

**Figure 9:**
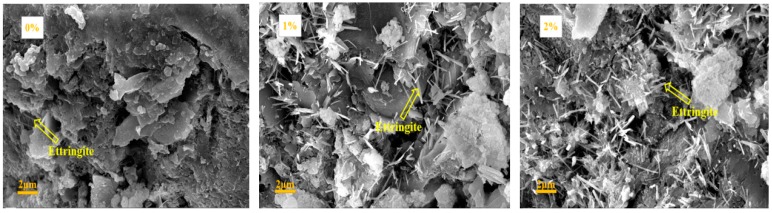

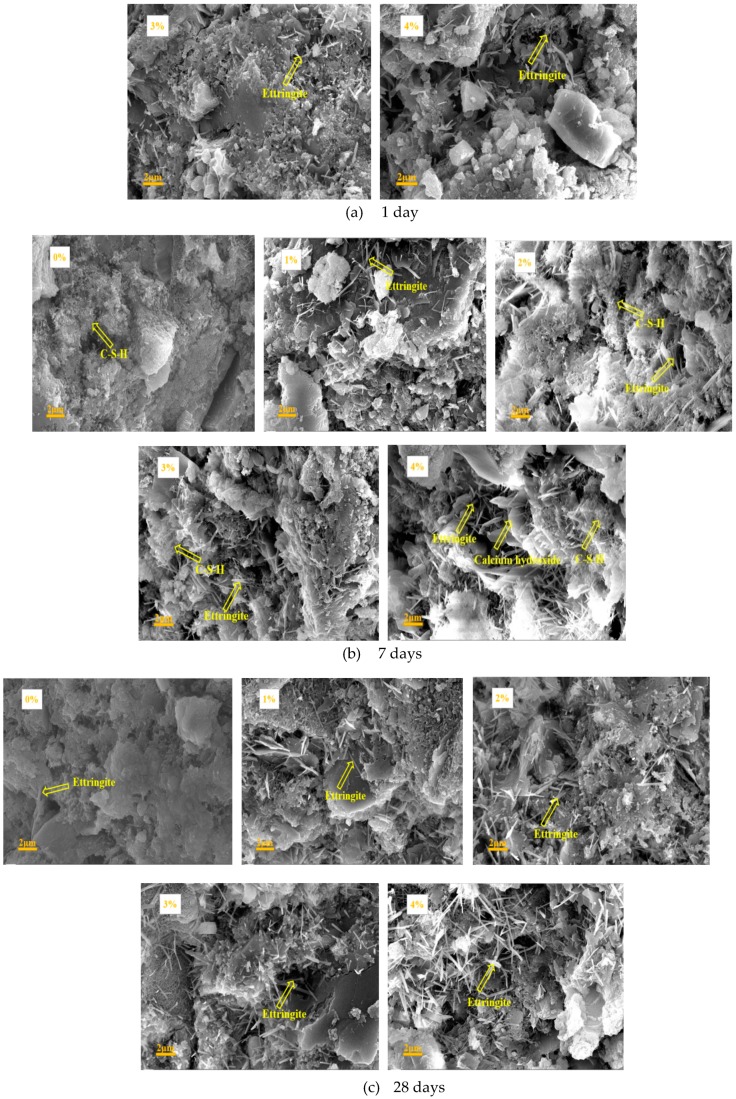
SEM results of the mixes with different contents of NaHCO_3_ at ages of (**a**) 1, (**b**) 7, and (**c**) 28 days.

The SEM results of the pastes with 0%, 1%, 2%, 3%, and 4% NaHCO_3_ at the ages of 1, 7, and 28 days are shown in Figure 9. At the age of 1 day, it can be seen that the amount of needle-shaped ettringite in the mixes with NaHCO_3_ was higher than that in the pure OPC paste. The microstructure of the C-S-H gel in the mixes with 1%, 2%, and 3% was denser than that in the pure OPC paste, but the C-S-H gel in the mix with 4% was a bit loose compared to the other groups. These agrees with the changing trend of the compressive strength with NaHCO_3_ at 1 day in Figure 2. At the age of 7 days, the mix with 1% NaHCO_3_ had more ettringite and denser C-S-H gel than the pure OPC paste, but further increase of NaHCO_3_ made the C-S-H gel become loose although the amount of ettringite was increased. At the age of 28 days, the ettringite in the mixes with NaHCO_3_ was still higher than that in pure OPC paste but the C-S-H gel in the mixes with NaHCO_3_ was looser than that in pure OPC paste. These agree with the results of XRD and compressive strength.

**Figure 10:**
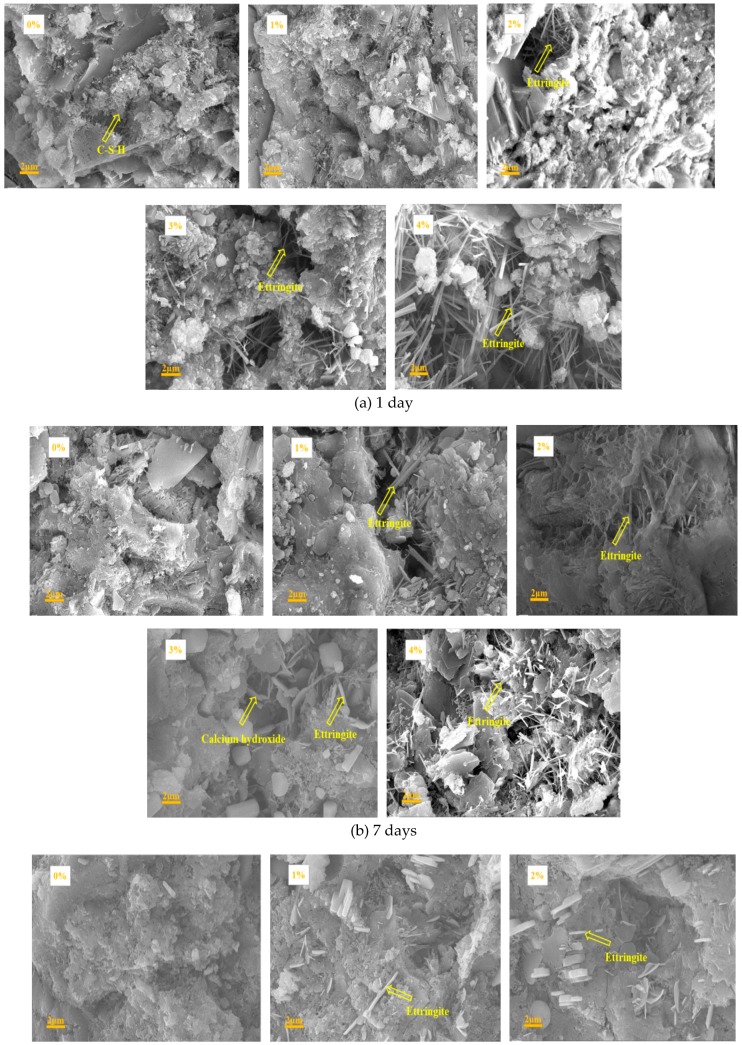

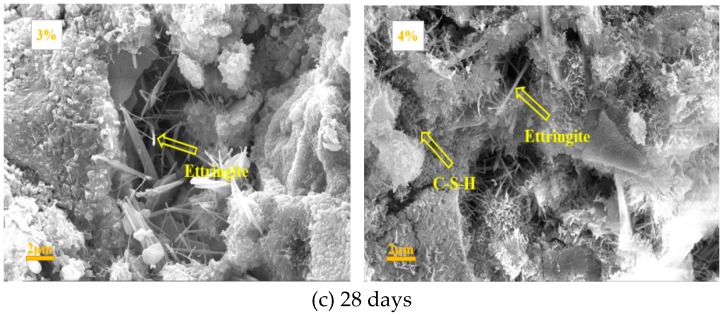
SEM results of the mixes with different contents of Na_2_CO_3_ at ages of (**a**) 1, (**b**) 7, and (**c**) 28 days.

Figure 10 shows the SEM images of the mixes with 0%, 1%, 2%, 3%, and 4% Na_2_CO_3_ at the ages of 1, 7, and 28 days. At the age of 1 and 7 days, the mix with 1% Na_2_CO_3_ had more ettringite and a denser C-S-H structure than the pure OPC paste. The mixes with 2–4% had more ettringite but a worse C-S-H gel structure than the pure OPC paste. At the age of 28 days, the C-S-H gel became worse with the increase of Na_2_CO_3_ content compared to the OPC paste with no Na_2_CO_3_. These results agree with the compressive strength results as shown in Figure 2. It could be indicated that the early age strength at 1 day was mainly influenced by both ettringite and C-S-H gel, and the later age strength, such as 28 days, was mainly influenced by C-S-H gel structure.

## 4. Discussion

### 4.1. Influence of Na_2_CO_3_/NaHCO_3_ on the PH of OPC Paste

**Figure 11:**
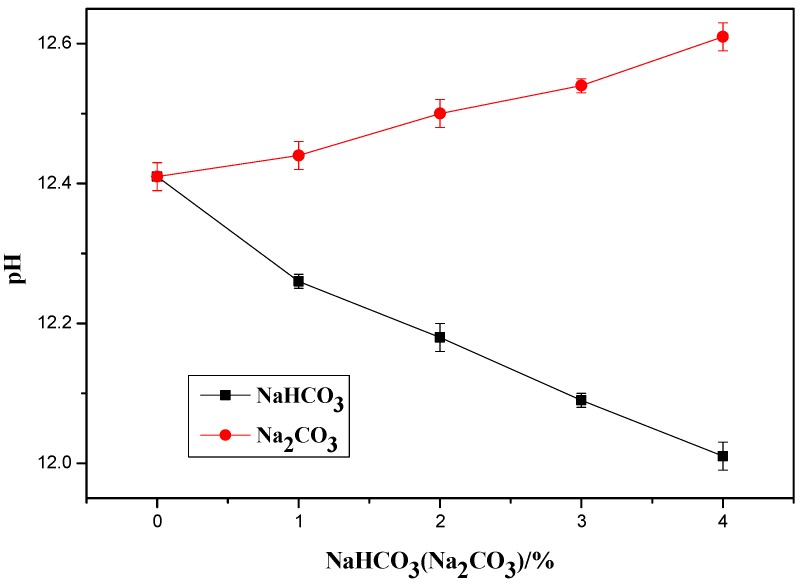
pH of fresh cement paste with Na_2_CO_3_ or NaHCO_3_.

It is known that the both Na_2_CO_3_ and NaHCO_3_ are soluble and their main difference is that the Na_2_CO_3_ dissolves into Na^+^ and CO_3_^2−^ and the NaHCO_3_ dissolves into Na^+^ and HCO_3_^−^ in water as shown in Equations (1) and (2). Solutions of Na_2_CO_3_ or NaHCO_3_ have a PH > 7, and the PH of Na_2_CO_3_ solution is higher than that of bicarbonate solution when the same content of the two are added. For example, under the same concentration 1 mmol/L (25 °C and 1 atm), the pH values of Na_2_CO_3_ and NaHCO_3_ solutions are 10.52 and 8.27 respectively. When they are added in cement paste, both of them can react with the portlandite, which is a hydration product of cement, and form CaCO_3_, as Equations (3) and (4). Cement slurry was prepared for pH measurements with a water-cement ratio of 0.5, a water reducing agent of 0.5%, and Na_2_CO_3_ and NaHCO_3_ of 0%, 1%, 2%, 3%, and 4%. The pH meter was initially calibrated with a neutral solution (pH = 7) and then with an alkaline solution with a known pH. After the calibration is completed, the electrode of the pH meter was immersed into the cement slurry and the slurry was gently vibrated to reach a uniform state during the measurements. The pH value was recorded after the reading was stable. The measured pH results are shown in Figure 11. It can be seen the pH of the OPC paste increased with the increase of Na_2_CO_3_ but it decreased with the increase of NaHCO_3_. This was caused by the different pH of the solutions with the same amount of Na_2_CO_3_ and NaHCO_3_. There could be a risk of alkali silica reaction in the concrete with a high amount of Na_2_CO_3_ because of the increased pH. There could be a decay of the C-S-H gel in the concrete with a high amount of NaHCO_3_ because of the decreased pH.

Na_2_CO_3_ → 2Na^+^ + CO_3_^2−^ (1)

NaHCO_3_ → Na^+^ + HCO_3_^−^ (2)

Na_2_CO_3_ + Ca(OH)_2_ = CaCO_3_ ↓ + 2NaOH (3)

NaHCO_3_ + Ca(OH)_2_ = NaOH + CaCO_3_ ↓ + H_2_O (4)

### 4.2. Influence of Na_2_CO_3_/NaHCO_3_ on the Introduced CO_2_

**Figure 12:**
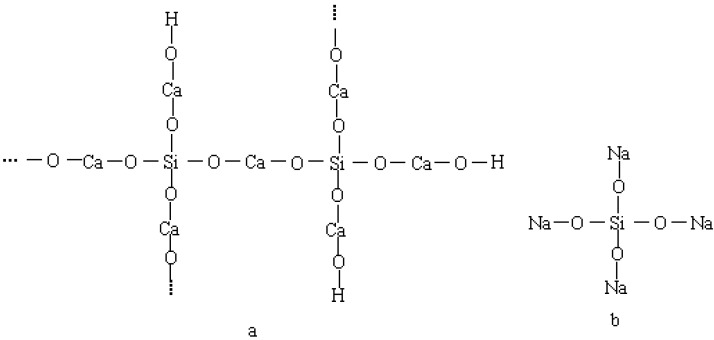
Sketch of the structure of C-S-H gel (**a**) and sodium orthosilicate (**b**).

In Na_2_CO_3_ the weight of Na^+^ is 43.4% and the weight of CO_2_ is 41.5%. In NaHCO_3_ the weight of Na^+^ is 27.4% and the weight of CO_2_ is 52.4%. It can be seen that, when the same weight of the two are used, NaHCO_3_ brings 16% less Na^+^ and 10.9% more CO_2_ into the cement paste. Although the Na^+^ is believed to accelerate the initial hydration and early age strength [37], it is thought that Na^+^ is responsible for the adverse effect on the later age strength development of the cement paste with salts containing Na^+^ [20]. The adverse effect of Na^+^ on the strength development can be explained that the Na^+^ could affect the adhesion between C-S-H gel structure (Figure 12a) by reaction with the silica phase in the cement paste and form sodium orthosilicate (Figure 12b) [38,39]. The difference of Na^+^ introduced by the two was thought to be the main reason that the paste with NaHCO_3_ had a better later stage strength development than that with Na_2_CO_3_.

### 4.3. Influence of Na_2_CO_3_/NaHCO_3_ on the Formation of Ettringite and CaCO_3_

The initial difference between the effects of the Na_2_CO_3_ and NaHCO_3_ on the setting time and early age strength can be explained by the following reasons. In the paste with Na_2_CO_3_ the initial reactions were as Equations (3) and (5)–(7), and there was formation of both CaCO_3_ and ettringite. While in the paste with NaHCO_3_ the initial reactions were as Equations (4), (5), and (7), and there was no initial formation of CaCO_3_ and there was only formation of ettringite. There could be less ettringite formed in the paste with Na_2_CO_3_ compared to that with NaHCO_3_ because of the initial consumption of Ca^2+^ with CO_3_^2−^. This could contribute to the better initial performance and shorter setting time of the paste with NaHCO_3_. In the paste with NaHCO_3_ the formation of CaCO_3_ happened at a later stage as Equations (8) and (9), and the later stage formed CaCO_3_ particles could contribute to fill the micro- and nano-pores of the C-S-H gel.

CaSO_4_•2H_2_O → Ca^2+^ + SO_4_^2−^ + 2H_2_O (5)

CO_3_^2−^ + Ca^2+^ → CaCO_3_ ↓ (6)

3CaO•Al_2_O_3_ + 3(CaSO_4_•2H_2_O) + 26H_2_O → 3CaO•Al_2_O_3_•3CaSO_4_•32H_2_O (7)

C_3_S + H_2_O → C-S-H + Ca^2+^ + OH^−^ (8)

HCO_3_^−^ + Ca^2+^ + OH^−^ → CaCO_3_ ↓ + H_2_O (9)

### 4.4. Influence of Na_2_CO_3_/NaHCO_3_ on the Enthalpies of the Reactions with C_3_S

In order to further investigate the effect of Na_2_CO_3_ and NaHCO_3_ on the hydration of C_3_S, the enthalpies of the reactions are calculated. The enthalpies of all the reactants and products were calculated by the first-principles and the module of total energy pseudopotential calculations in the Vienna Ab initio Simulation Package (VASP) [40] was used for the calculations.

2(3CaO·SiO_2_) + 6H_2_O = 3CaO·2SiO_2_·3H_2_O + 3Ca(OH)_2_ (10)

Ca(OH)_2_ + Na_2_CO_3_ = CaCO_3_ + 2NaOH (11)

Ca(OH)_2_ + NaHCO_3_ = CaCO_3_ + NaOH + H_2_O (12)

3CaO·2SiO_2_·3H_2_O + 2NaOH = Na_2_O·2CaO·2SiO_2_·H_2_O + Ca(OH)_2_ + H_2_O (13)

By combing Equations (10), (11) and (13), resulting Equation (14)

2(3CaO·SiO_2_) + Na_2_CO_3_ + 4H_2_O = Na_2_O·2CaO·2SiO_2_·H_2_O + CaCO_3_ + 3Ca(OH)_2_ (14)

By combing Equations (10), (12) and (13), resulting Equation (15)

2(3CaO·SiO_2_) + 2NaHCO_3_ + 2H_2_O = Na_2_O·2CaO·2SiO_2_·H_2_O + 2CaCO_3_ + 2Ca(OH)_2_ (15)

The enthalpy of the reactions in Equation (14) (E_reaction14_) can be calculated by Equation (16) and the value was −0.02903 eV/atom. The enthalpy of the reactions in Equation (15) (E_reaction15_) can be calculated by Equation (17) and the value was −0.04306 eV/atom. These negative values suggest that the reactions in Equations (14) and (15) are both exothermic and can proceed spontaneously in thermodynamics. The reaction in Equation (15) had a more negative value than that in Equation (14) and it means that the reaction in Equation (15) is much easier to happen than that in Equation (14), which suggests that the reaction between C_3_S and NaHCO_3_ is much easier that the reaction between C_3_S and Na_2_CO_3_.

E_reaction14_ = (E_Na2O·2CaO·2SiO2·H2O_ × 16 + E_CaCO3_ × 5 + E_Ca(OH)2_ × 15-E_3CaO·SiO2_ × 18-E_Na2CO3_ × 6-E_H2O_ × 12)/36 (16)

E_reaction15_ = (E_Na2O·2CaO·2SiO2·H2O_ × 16 + E_CaCO3_ × 10 + E_Ca(OH)2_ × 10-E_3CaO·SiO2_ × 18-E_NaHCO3_ × 18-E_H2O_ × 6)/36 (17)

where E_3CaO·SiO2_, E_Na2CO3_, E_NaHCO3_, E_H2O_, E_Na2O·2CaO·2SiO2·H2O_, E_CaCO3_, E_Ca(OH)2_ are the enthalpies of 3CaO·SiO_2_, Na_2_CO_3_, NaHCO_3_, H_2_O, Na_2_O·2CaO·2SiO_2_·H_2_O, CaCO_3_, and Ca(OH)_2_ molecules in unit of eV/atom, respectively.

## 5. Conclusions

The influence of NaHCO_3_ and Na_2_CO_3_ as additional additives on the setting time and compressive strength of OPC paste was investigated and the related effect on the hydration mechanism was studied through TG-DTA, XRD, and SEM tests. The following conclusions can be drawn.

(1) The initial and final setting time of OPC paste decreased with the increase of either NaHCO_3_ or Na_2_CO_3_.
(2) The addition of either NaHCO_3_ or Na_2_CO_3_ could increase the early age compressive strength (1 and 7 days) depending on the content added but they could decrease the compressive strength at later ages, such as 28 days, with the increase of content added.
(3) As an accelerator, the optimum content of NaHCO_3_ and Na_2_CO_3_ were found to be in the same level as 1% of the weight of OPC. The addition 1% of either of the two accelerators could significantly shorten the setting time, increase the early age strength and did not have an obvious detrimental effect on the later age strength.
(4) Further increase of NaHCO_3_ and Na_2_CO_3_ above 1% could decrease the compressive strength of OPC paste although the ettringite formation was accelerated and increased. This decay was mainly caused by the Na^+^ ions introduced and the Na^+^ could partly replace the Ca^2+^ in the C-S-H gel and cause the discontinuity of the C-S-H gel.
(5) NaHCO_3_ was seen to be a better option as an accelerator compared to Na_2_CO_3_. The reaction between NaHCO_3_ and C_3_S was found to be much easier than the reaction between Na_2_CO_3_ and C_3_S. The same amount addition of NaHCO_3_ resulted a higher compressive strength at all ages compared to NaHCO_3_. Besides, NaHCO_3_ the introduced less Na^+^ and more CO_2_ in the cementitious system than the Na_2_CO_3_ when the same amount of the two were used.

## Figures and Tables

**Table 1:** Physical properties of ordinary Portland cement (OPC).

Fineness/%	Stability	Setting Time/min		Flexural Strength/MPa		Compressive Strength/MPa	
		Initial	Final	3 days	28 days	3 days	28 days
1.5	Qualified	181	378	5.1	9.3	25.3	51.6

**Table 2:** Chemical composition of OPC/%.

SiO_2_	CaO	Al_2_O_3_	Fe_2_O_3_	MgO	Na_2_O	K_2_O	LOI
22.96	63.87	5.73	3.31	2.64	0.32	0.23	0.18

**Table 3:** Mineral composition of OPC/%.

C_3_S	C_2_S	C_3_A	C_4_AF
54.5	19.23	8.36	10.14

**Table 4:** Mix design.

Mix ID	OPC/%	Superplasticizer/%	NaHCO_3_/%	Na_2_CO_3_/%	W/C
1	100	0.5	0	0	0.35
2			1	-	
3			2	-	
4			3	-	
5			4	-	
6			-	1	
7			-	2	
8			-	3	
9			-	4	

## References

[1] Ma B., Ma M., Shen X., Li X., Wu X. (2014). Compatibility between a polycarboxylate superplasticizer and the belite-rich sulfoaluminate cement: Setting time and the hydration properties. Constr. Build. Mater..

[2] Bamonte P., Gambarova P.G., Nafarieh A. (2016). High-temperature behavior of structural and non-structural shotcretes. Cem. Concr. Compos..

[3] De Belie N., Grosse C.U., Kurz J., Reinhardt H. (2005). Ultrasound monitoring of the influence of different accelerating admixtures and cement types for shotcrete on setting and hardening behaviour. Cem. Concr. Res..

[4] Won J., Choi B., Lee J. (2012). Experimental and statistical analysis of the alkali–silica reaction of accelerating admixtures in shotcrete. Constr. Build. Mater..

[5] Xie J., Wang J., Rao R., Wang C., Fang C. (2019). Effects of combined usage of GGBS and fly ash on workability and mechanical properties of alkali activated geopolymer concrete with recycled aggregate. Compos. B Eng..

[6] Hou P., Kawashima S., Kong D., Corr D.J., Qian J., Shah S.P. (2013). Modification effects of colloidal nano SiO_2_ on cement hydration and its gel property. Compos. B Eng..

[7] Meng T., Yu Y., Wang Z. (2017). Effect of nano-CaCO3 slurry on the mechanical properties and micro-structure of concrete with and without fly ash. Compos. B Eng..

[8] Nazari A., Riahi S. (2011). The effects of zinc dioxide nanoparticles on flexural strength of self-compacting concrete. Compos. B Eng..

[9] Jalal M., Ramezanianpour A.A., Pool M.K. (2013). Split tensile strength of binary blended self compacting concrete containing low volume fly ash and TiO_2_ nanoparticles. Compos B Eng..

[10] Brough A.R., Atkinson A. (2002). Sodium silicate-based. alkali-activated slag mortars: Part I. Strength, hydration and microstructure. Cem. Concr. Res..

[11] Li G., Li C., Zhou W., Wu Y. (2005). Factors affecting the liquid sodium aluminate accelerated agent. Concrete.

[12] Han J., Wang K., Shi J., Wang Y. (2014). Influence of sodium aluminate on cement hydration and concrete properties. Constr. Build. Mater..

[13] Tu Z., Shi C., Farzadnia N. (2018). Effect of Limestone Powder Content on the Early-Age Properties of CO_2_-Cured Concrete. J. Mater. Civ. Eng..

[14] Chong L., Shi C., Yang J., Jia H. (2017). Effect of limestone powder on the water stability of magnesium phosphate cement-based materials. Constr. Build. Mater..

[15] Wu Z., Shi C., Khayat K.H. (2018). Multi-scale investigation of microstructure, fiber pullout behavior, and mechanical properties of ultra-high performance concrete with nano-CaCO_3_ particles. Cem. Concr. Compos..

[16] Li W., Huang Z., Zu T., Shi C., Duan W.H., Shah S.P. (2016). Influence of Nanolimestone on the Hydration, Mechanical Strength, and Autogenous Shrinkage of Ultrahigh-Performance Concrete. J. Mater. Civ. Eng..

[17] Tu Z., Guo M., Poon C.S., Shi C. (2016). Effects of limestone powder on CaCO_3_ precipitation in CO_2_ cured cement pastes. Cem. Concr. Compos..

[18] Monosi S., Moriconi G., Collepardi M. (1982). Combined effect of lignosulfonate and carbonate on pure portland clinker compounds hydration. III. Hydration of tricalcium silicate alone and in the presence of tricalcium aluminate. Cem. Concr. Res..

[19] Krismahariyanto M., Saing B., Widodo H. (2017). The Effects of Sodium Carbonate on the Properties and the Hydration of the Cement Mixed with the Hardening Accelerator Based on Calcium-Aluminate. AIP Conf. Proc..

[20] Jang J.G., Kim H.J., Park S.M., Lee H.K. (2015). The influence of sodium hydrogen carbonate on the hydration of cement. Constr. Build. Mater..

[21] Yang H., Che Y. (2018). Effects of Nano-CaCO_3_/Limestone Composite Particles on the Hydration Products and Pore Structure of Cementitious Materials. Adv. Mater. Sci. Eng..

[22] Bernardi A., Bortoluzzi E.A., Felippe W.T., Felippe M.C.S., Wan W.S., Teixeira C.S. (2017). Effects of the addition of nanoparticulate calcium carbonate on setting time, dimensional change, compressive strength, solubility and pH of MTA. Int. Endod. J..

[23] Mathur R., Sharma S.K. (2008). Magnesium oxysulphate cement: Change in properties on admixing sodium bicarbonate as an additive. Rasayan J.Chem..

[24] Chandrawat M.P.S., Yadav R.N. (2001). Effect of sodium carbonate on some properties of magnesia cement. J. Indian Chem. Soc..

[25] Kunther W., Lothenbach B., Scrivener K. (2013). Influence of bicarbonate ions on the deterioration of mortar bars in sulfate solutions. Cem. Concr. Res..

[26] Kerui Y., Caiwen Z., Zhigang L. (2002). The influence of calcium lignosulphonate–sodium bicarbonate on the status of ettringite crystallization in fly ash cement paste. Cem. Concr. Res..

[27] Reddy V.V., Rao H.S., Jayaveera K.N. (2006). Influence of strong alkaline substances (sodium carbonate and sodium bicarbonate) in mixing water on strength and setting properties of concrete. Indian J. Eng. Mater. Soc..

[28] Reddy L.V.G., Krishna B. (2014). Influence of Na_2_CO_3_, NaHCO_3_ and Organic Substances (Algae) in Water on Physical Properties of Blended Pozzolonic Portland Cement. Int. J. Eng. Res. Technol. (IJERT)..

[29] Golewski G.L. (2018). Evaluation of morphology and size of cracks of the Interfacial Transition Zone (ITZ) in concrete containing fly ash (FA). J. Hazard. Mater..

[30] Golewski G.L. (2018). An assessment of microcracks in the Interfacial Transition Zone of durable concrete composites with fly ash additives. Compos. Struct..

[31] Standardization Administration of the People’s Republic of China (2007). GB175-2007: Common Portland Cement.

[32] Ministry of Housing and Urban-Rural Development (2005). JC 477-2005 Flash Setting Admixtures for Shotcrete.

[33] Zhang Y., Wang Y., Li T., Xiong Z., Sun Y. (2018). Effects of lithium carbonate on performances of sulphoaluminate cement-based dual liquid high water material and its mechanisms. Constr. Build. Mater..

[34] Xiong Z., Wang P., Wang Y. (2015). Hydration Behaviors of Portland Cement with Different Lithologic Stone Powders. Int. J. Concr. Struct. Mater..

[35] Bensted J., Barnes P. (2008). Structure & Performance of Cements.

[36] Bullard J.W., Jennings H.M., Livingston R.A., Nonat A., Scherer G.W., Schweitzer J.S., Scrivener K.L., Thomas J.J. (2011). Mechanisms of cement hydration. Cem. Concr. Res..

[37] Edwards G.C., Angstadt R.L. (2007). The effect of some soluble inorganic admixtures on the early hydration of portland cement. J. Appl. Chem..

[38] Abdalqader A.F., Jin F., Al-Tabbaa A. (2016). Development of greener alkali-activated cement: Utilisation of sodium carbonate for activating slag and fly ash mixtures. J. Clean. Prod..

[39] Yuan B., Straub C., Segers S., Yu Q.L., Brouwers H.J.H. (2017). Sodium carbonate activated slag as cement replacement in autoclaved aerated concrete. Ceram. Int..

[40] Kresse G., Furthmuller J. (1996). Efficient iterative schemes for ab initio total-energy calculations using a plane-wave basis set. Phys. Rev. B.

